# Spatiotemporal Changes in Frost-Free Season and Its Influence on Spring Wheat Potential Yield on the Qinghai–Tibet Plateau from 1978 to 2017

**DOI:** 10.3390/ijerph20054198

**Published:** 2023-02-26

**Authors:** Zemin Zhang, Changhe Lu

**Affiliations:** 1 State Key Laboratory of Environmental Criteria and Risk Assessment, Chinese Research Academy of Environmental Sciences, Beijing 100012, China; 2 Institute of Ecology, Chinese Research Academy of Environmental Sciences, Beijing 100012, China; 3 Key Laboratory of Land Surface Pattern and Simulation, Institute of Geographical Sciences and Natural Resources Research (CAS), Beijing 100101, China; 4 College of Resources and Environment, University of Chinese Academy of Sciences, Beijing 100049, China

**Keywords:** frost-free season, change trend, spring wheat, Qinghai–Tibet Plateau

## Abstract

Accurately assessing the variation in the frost-free season (FFS) can provide decision support for improving agricultural adaptability and reducing frost harm; however, related studies were inadequate in terms of the Qinghai–Tibet Plateau (QTP). This study analyzed the spatiotemporal changes in the first frost day in autumn (FFA), last frost day in spring (LFS), FFS length and effective accumulated temperature (EAT) during the 1978–2017 period, and their influences on spring wheat potential yield on the QTP, based on daily climatic data and the methodology of Sen’s slope and correlation analysis. The results showed that the annual average FFA and LFS occurred later and earlier from northwest to southeast, respectively, and both the FFS length and EAT increased. From 1978 to 2017, the average regional FFA and LFS were delayed and advanced at rates of 2.2 and 3.4 days per decade, and the FFS and EAT increased by 5.6 days and 102.7 °C·d per decade, respectively. Spatially, the increase rate of FFS length ranged from 2.8 to 11.2 days per decade throughout the QTP, and it was observed to be larger in northern Qinghai, central Tibet and Yunnan, and smaller mainly in eastern Sichuan and southern Tibet. Correspondingly, the increase rate for EAT ranged from 16.2 to 173.3 °C·d per decade and generally showed a downward trend from north to south. For a one-day increase in the FFS period, the spring wheat potential yield would decrease by 17.4 and 9.0 kg/ha in altitude ranges of <2000 m and 2000–3000 m, but decrease by 24.9 and 66.5 kg/ha in the ranges of 3000–4000 m and >4000 m, respectively. Future studies should be focused on exploring the influence of multiple climatic factors on crop production using experimental field data and model technologies to provide policy suggestions.

## 1. Introduction

The world is experiencing climate change characterized by a warming trend. According to the sixth assessment report of the Intergovernmental Panel on Climate Change (IPCC), the global average surface temperature increased by 1.07 °C from the 1850–1900 period to the 2010–2019 period, resulting in a continuous increase in the frequency and intensity of extreme weather events, with serious and widespread impacts on humans, non-human species and the environment [1]. As an important field related to food security, agricultural production is becoming vulnerable and facing some challenges under the current trend of climate change [2]. The Qinghai–Tibet Plateau (QTP) is an important indicator of global climate change, and its warming trend is more remarkable [3,4]. From 1965 to 2013, the average temperature increased by around 2.5 °C on the QTP, 2.4 folds higher than that in the whole of China, which has had a profound influence on local agricultural production and resource development [5].

The frost-free season (FFS) is an important index that reflects climate change. Accurately identifying its variation can guide farmers in adjusting crop-sowing and livestock stocking dates and serves as a key consideration for introducing adaptive crop varieties [6,7,8]. For instance, a delayed first frost day in autumn (FFA) could reduce the possibility of frost disaster to crops, while advancing the last frost date in spring (LFS) is beneficial for adjusting the sown date of crops [9]. Therefore, attention should be paid to the regional change characteristics of FFS in the study of agricultural adaptability against the warming trend [10,11,12,13]. However, studies regarding the influence of FFS on spring wheat potential yield on the QTP have not been reported.

At present, several studies have reported worldwide changes in the FFS, and the studied areas mainly cover the United States [14], Canada, Germany [15], northeastern Australia [7] and Central Europe [16], as well as the whole world [17]. These studies found that the FFS has lengthened in the last century; for instance, the FFA was obviously delayed in the United States, while the LFS showed a tendency to advance, and the latter contributed more to the increased number of FFS days [6]. In China, more than 80% of the land area has shown trends of delayed FFA, advanced LFS and prolonged FFS since 1951 [18,19]. The FFA occurred later, at an average rate of 1.8 days per decade in northwestern China [20,21]; specifically, it has been delayed by 11.0, 7.0 and 5.5 days in Xinjiang, Shaanxi Provinces and the Hengduan Mountain area, respectively, since 1960 [21,22,23,24]. Regarding the LFS, the areas with larger delay rates were concentrated in northwestern, central and eastern China, while the western and southern regions had larger advance rates [8]. Under the influence of a delayed FFA and advanced LFS, the FFS differed in different regions. In Shanxi, Shaanxi, the northwest and northeast regions, the FFA showed a tendency to be prolonged [25,26]. However, both the FFA and LFS occurred later in Chongqing; nevertheless, FFS was lengthened due to the larger delay rate of the FFA [27].

On the QTP, the regional FFA, LFS and FFS also showed trends of delaying, advancing and lengthening, respectively [8,9,10,11,28,29]. Ning et al. analyzed the spatial distribution of the FFA, LFS and FFS for the whole of China in the 1951–2011 period using the IDW interpolation method and found that the FFS was lengthened and that the FFA and LFS were delayed and advanced, respectively, on the QTP [8]. Zhang et al. investigated the changes in FFA, LFS and FFS across 73 meteorological sites on the QTP and obtained similar results [9]. Li et al. also analyzed the changing trend in the FFS in China based on the ERA5-Land dataset but did not involve results relating to the FFA and LFS on the QTP [29]. Overall, these studies mainly focused on specific sites or the whole of China, and the spatial distribution was mapped using ordinary interpolation methods. Considering that the typical landscape features of high altitude and the complex terrain on the QTP could have obvious effects on regional FFS, the accuracy of the obtained results based on ordinary interpolation methods is unknown.

In this study, we analyzed the changing trends of the FFA, LFS, FFS and effective accumulated temperature (EAT) in the FFS based on daily temperature records taken from 131 meteorological sites on the QTP from 1978 to 2017. Then, its spatiotemporal changes in relation to altitude were explored. Finally, the driving factors of FFS change and its influence on spring wheat potential yield in different altitudes and the whole QTP were analyzed.

## 2. Study Area

**Figure 1:**
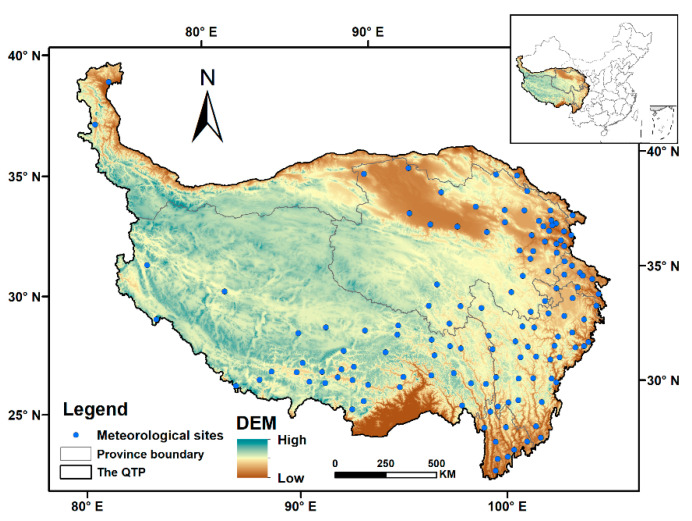
Location and spatial distribution of meteorological sites on the Qinghai–Tibet Plateau.

The QTP (26°20′–39°30′ N, 73°20′–104°20′ E) is located in western China and covers the whole Tibet Autonomous Region and Qinghai Province and parts of Sichuan, Gansu, Yunnan and Xinjiang provinces. Known as the “Roof of the World”, the “Third pole” and the “Water Tower of Asia”, the QTP is the highest geographic unit on Earth and has an average altitude of over 4000 m, with the altitude rising from the southeast and northeast to the central and western regions (Figure 1). On the QTP, the alpine climate characteristics are very obvious, with low surface temperatures and large diurnal ranges. The temperature decreases significantly with increasing altitude and latitude, and the average temperature gradually decreases from southeast to northwest. In the northern QTP, the annual average temperature is mostly below 5.0 °C and over 9.0 °C in the southern region [30]. Its agricultural planting system is dominated by single-season crops, and the primary grain crops are spring wheat, highland barley and peas, normally grown from early April to September [31].

## 3. Data and Methods

### 3.1. Data Sources and Preprocessing

The daily temperature records were sourced from the data center of resources and environment science of the Chinese Academy of Sciences “http://www.resdc.cn/Default.aspx”(accessed on 6th May 2021), including average, maximum and minimum temperatures across 131 sites on the QTP in the 1978–2017 period. The format of the original file is .TXT. The daily climatic data recorded on a monthly basis were stored in a file, and the unit of temperature was 0.1 °C; therefore, the values of the original data were multiplied by 0.1 before further processing using Python 3.6.7 (Python Software Foundation, Wilmington, DE, United States) based on the PyCharm Communication Edition platform. To improve the accuracy of calculation, the missing daily records of a few sites were filled using the mean values of the preceding and following four days and the sites with the missing days equal to or above 10 days in one year were eliminated.

The potential yield of spring wheat was simulated based on daily climate data and the validated WOFOST model (WOFOST 7.1.7 version, Wageningen University, Wageningen, Netherlands) across 113 meteorological sites, where it was suitable for planting spring wheat on the QTP. More details regarding the simulation and validation of the potential yield of spring wheat can be found in reference [31].

### 3.2. Calculation of FFS and EAT at the Site Level

The indices of LFS, FFA and FFS were determined using the inclusive threshold of 0 °C for the daily minimum temperature. In an agricultural field, the dates when the ground temperature was below 0 °C for the first time in autumn and for the last time in spring were called the FFA and LFS (Julian day) [32]. However, due to the fact that the temperature is prone to repeated fluctuations across seasonal dates, it is very likely that the daily minimum temperature would return to be above or below 0 °C after becoming lower or higher than 0 °C for the first time; therefore, it may not be accurately used to identify the dates of the LFS and FFA only according to the first time when the daily minimum temperature is below or above 0 °C. Therefore, we selected the first day when the daily minimum temperature was above 0 °C for five consecutive days for the first time and the last day when it was above 0 °C for five consecutive days for the last time as the LFS and FFA of each site. The number of days between LFS and FFA was identified as the FFS length. Then, the value of EAT was calculated according to daily maximum and minimum temperatures during the FFS period (Equation (1)).

(1) $$EAT=\sum_{LFS}^{FFF}\left[\left(T_{max}+T_{min}\right)/2-T_{b}\right]$$

where *EAT* indicates the effective accumulated temperature; *FFA* and *LFS* denote the first frost day in autumn and last frost day in spring (Julian day), respectively; *T_max_* and *T_min_* are the daily maximum and minimum temperature; and *T_b_* is the base temperature, which is 0 °C here.

### 3.3. Estimation of Change Rate

In this study, Sen’s SLOPE method was used to quantitatively analyze the annual change rates of FFA, LFS, FFS and EAT for each site in the 1978–2017 period. It is a nonparametric estimation method used for the linear regression of sequence data [33,34]. The general forms are expressed below as follows:

(2) Y(t)=SLOPE·t+b

(3) $$SLOPE_{i}=\frac{Y_{j}-Y_{k}}{j-k}\;$$

where *b* is the constant term, *i* denotes the county, *j* and *k* denote years (*j* > *k*), *SLOPE_i_* is the Sen’s slope value of the yield changes for county *i*, and *Y_j_* and *Y_k_* are the maize yield in year *j* and *k*, respectively. When N is allowed to be the time series length, *SLOPE* could be presented below as follows:

(4) $$SLOPE=\{\begin{array}{c} SLOPE_{\frac{\left(N+1\right)}{2}}\;\;\;\;\;\;\;\;\;\;\;\;\;\;\;\;\;\;\;\;\;\;\;\;\;\;\;\;\;\;\;\;\;\;\;\;\;\;\;N\;\mathrm{is}\;\mathrm{odd}\\ \left(SLOPE_{\frac{N}{2}}+SLOPE_{\frac{\left(N+2\right)}{2}}\right)/2\;\;\;\;\;\;\;N\;\mathrm{is}\;\mathrm{even}\; \end{array}$$

In this study, the Excel template application MAESENS of the Mann–Kendall and Sen’s slope methods, as developed by Salmi et al. [34], was used to identify the change rates of FFA, LFS, FFS and EAT for each station in the 1978–2017 period.

### 3.4. Correlation Analysis

The influence of FFS on changes to the potential yield of spring wheat was analyzed using correlation analysis with the following calculation formula:

(5) $$R=\frac{\sum_{i=1}^{N}X_{i}Y_{i}-\frac{\sum_{i=1}^{N}X_{i}\sum_{i=1}^{N}Y_{i}}{N}}{\sqrt{\left[\sum_{i=1}^{N}X_{i}^{2}-\frac{{\left(\sum_{i=1}^{N}X_{i}\right)}^{2}}{N}\right]\left[\sum_{i=1}^{N}Y_{i}^{2}-\frac{{\left(\sum_{i=1}^{N}Y_{i}\right)}^{2}}{N}\right]}}\;\;\;$$

where *R* is the correlation coefficient, *X_i_* and *Y_i_* represent the FFS days and the potential yield of spring wheat in the year *i*, and *N* is the number of years. When *R* > 0, these two variables are positively correlated, while when *R* < 0, they are negatively correlated. A larger |*R*| indicates a stronger correlation between them.

## 4. Results

### 4.1. Statistical Analyses of FFS and EAT

**Figure 2:**
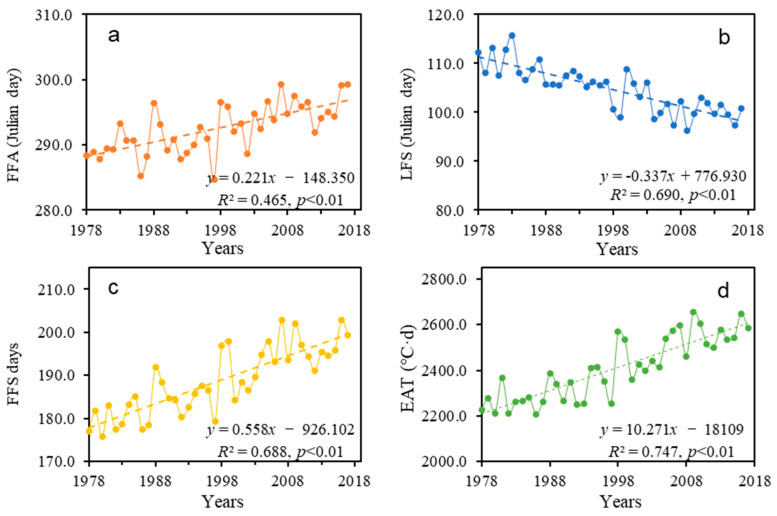
Changes in FFA (**a**), LFS (**b**), FFS (**c**) and EAT (**d**) on the Qinghai–Tibetan Plateau from 1978 to 2017.

The annual averages of the FFA and LFS were 293 (20th October) and 105 (15th April), respectively, at all sites across the QTP, and the FFS length was an average of 188 days. From 1978 to 2017, the FFA, LFS and FFS across the whole of the QTP showed an obvious change trend: the FFA was delayed from 288 (15th October) in 1978 to 297 (24th October) in 2017 at a significant rate of 2.2 days per decade (*R*^2^ = 0.465, *p* < 0.01) (Figure 2a). The LFS advanced by 13 days cumulatively after 1978, at a rate of 3.4 days per decade (*R*^2^ = 0.690, *p* < 0.01) (Figure 2b). Under the influence of a delayed FFA and an advanced LFS, the length of the FFS increased significantly, at 5.6 days per decade (*R*^2^ = 0.688, *p* < 0.01), with a cumulative increase of 23 days (Figure 2c). The EAT in FFS increased from 2225.8 to 2587.2 °C·d at an upward rate of 102.7 °C per decade (Figure 2d).

At the site level, from 1978 to 2017, the annual average FFA and LFS, respectively, ranged from 249 (6th September) and 4 (4th January) to the dates of 363 (29th December) and 159 (8th June). Accordingly, the FFS was between 92 and 361 days, and the EAT was between 850.8 and 5798.8 °C·d. Regarding this trend, the change rate of FFA ranged from −1.4 to 10.2 days per decade across all sites, among which 119 sites showed a delayed trend. The change rate of LFS ranged from −1.5 to 2.0 days per decade, and as many as 126 stations showed an advancing trend. For the FFS and EAT, all stations showed increasing trends, with the highest increase rates of 11.2 days and 171.2 °C d per decade, respectively.

**Figure 3:**
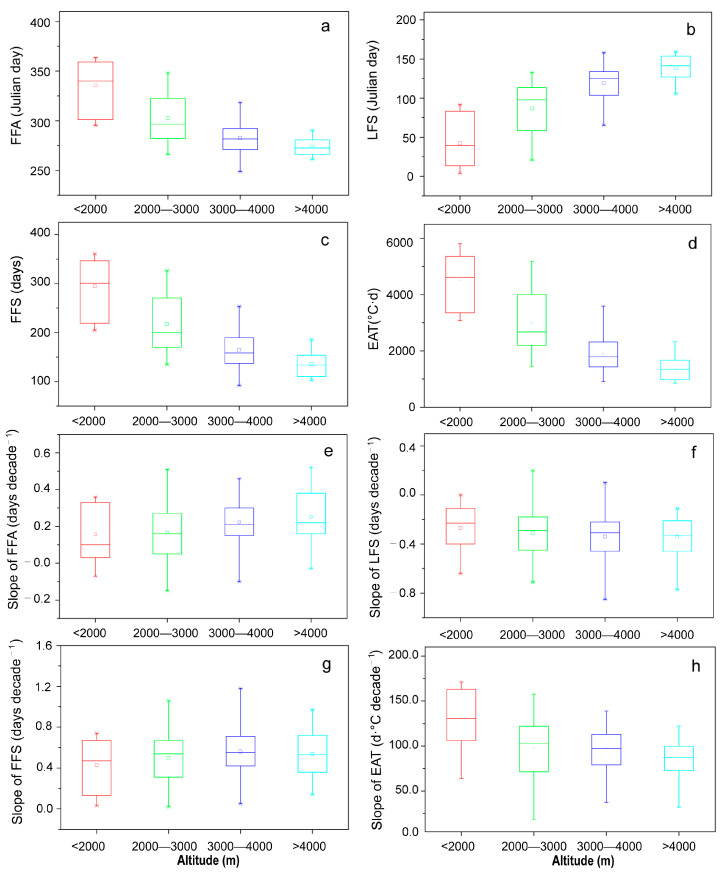
Statistical analysis of the first and last frost days and frost-free periods and their change rates in different altitude ranges on the Qinghai–Tibetan Plateau. a–d represent annual mean values of FFA, LFS, FFS and EAT in different altitude ranges; e–h represent annual change rates of FFA, LFS, FFS and EAT in different altitude ranges. Note: The upper, middle and lower lines in the box indicate the upper quartile, median and lower quartile values of climatic factors and their change rates at all stations in each accumulated temperature range, respectively. The – above and below indicates the maximum and minimum values, × on and above and below indicates the 1% and 99% values, and □ represents the mean value at all stations.

To further analyze the change characteristics of the above indices at different altitudes, we classified all of the sites into four altitude ranges, including <2000, 2000–3000, 3000–4000 and >4000 m, covering 10, 47, 53 and 21 sites, respectively. The results showed that the FFA and LFS generally occurred earlier and later, respectively, with the increasing altitude, while FFS length and EAT decreased. For instance, in the range of <2000 m, the average FFS was 294 days, 158 days longer than that in the range of >4000 m. The FFA and LFS in the range of <2000 m occurred on 335 (1st December) and 43 (12th February), respectively, 61 and 96 days later and earlier than those above 4000 m (Figure 3a–c). Additionally, the annual average EAT declined from 4508.2 at sites in the <2000 m range to 1379.5 °C·d at a range of >4000 m (Figure 3d).

Regarding the inter-annual change, the delay rate of the FFA increased from 1.5 to 2.5 days per decade from the range of <2000 m to >4000 m, and the advancing rate of the LFS increased from 2.7 to 3.4 days per decade. As a result, the increase rate of FFS length increased from 4.3 to 5.9 days per decade. However, the increase rate of EAT decreased from 130.7 to 83.8 °C·d per decade. In total, with increased altitude, the delay rate of FFA, the advance rate of LFS and the rate of FFS length increase generally showed an increasing trend; nevertheless, the rate of EAT increase displayed a decreasing trend, which was mainly caused by decreased temperature and shortened FFS length (Figure 3e–h).

### 4.2. Spatiotemporal Changes in FFS and EAT

**Figure 4:**
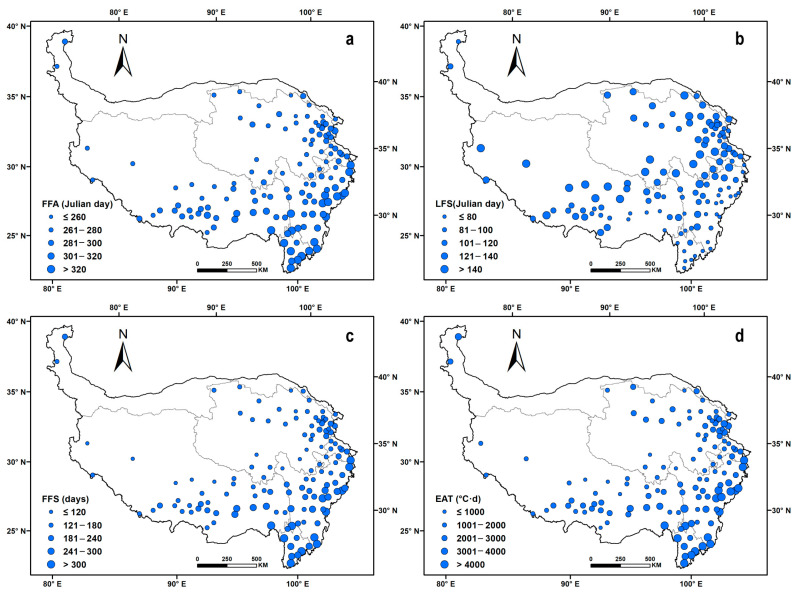
Annual mean values of the first and last frost days (Julian days) and frost-free periods on the Qinghai–Tibetan Plateau in the 1978–2017 period. a–d represent the spatial distribution of annual mean values of FFA, LFS, FFS and EAT at the site level.

The annual average FFA ranged from 249 (6th September) to 365 (31st December) across the QTP, and it occurred later, gradually moving from northwest to southeast. Specifically, the FFA occurred prior to the date of 280 (17th September) at 47 sites, which were mainly located in northern Qinghai and northeastern Tibet and the southern regions with higher altitudes. In eastern Qinghai, and the southern areas of Tibet, Sichuan and Gansu, the FFA ranged from 281 (18th September) to 320 (16th December). At the other 20 sites, distributed in southern Tibet, Yunnan and Sichuan, the FFA generally occurred after 320 (16th December) (Figure 4a).

Contrary to FFA, the LFS gradually occurred earlier, moving from northwest to southeast at the site level. The sites with an LFS prior to the date of 100 (10th April) were mainly located in the southern QTP. Secondly, in the Qaidam Basin and the plateau area of Xinjiang, the LFS was between the dates of 100 (10th April) and 120 (30th April). The sites ranging from the date of 121 (1st May) to 140 (20th May) were mostly distributed in the low-altitude areas of southern Tibet. The sites with an LFS occurring after 140 (20th May) were mainly located in the junction regions of Tibet and Qinghai provinces and some other areas scattered on the border of Qinghai, Gansu provinces and high-altitude areas of southern Tibet (Figure 4b).

Regarding the FFS, its length showed a gradually increasing trend from northwest to southeast. Twelve sites with fewer than 120 days were mainly concentrated in western Tibet and southern Qinghai, and 26 sites with longer than 240 days were mainly located in the southwestern QTP, such as southern Tibet, Sichuan and Yunnan. At the other 93 sites, the length of the FFS ranged from 120 to 240 days (Figure 4c). Accordingly, the annual average EAT gradually increased from the northwest to the southeast. Spatially, there were 58 sites with an EAT below 2000 °C·d, and they were widely distributed in the hinterland of the plateau, including most regions of Tibet and Qinghai province. Overall, 54 sites between 2000 and 4000 °C·d were mostly distributed in large regions of central Tibet, eastern Qinghai and northern Sichuan. In southern Tibet and some canyons in Yunnan and Sichuan (19 sites), the EAT was generally above 4000 °C·d (Figure 4d).

**Figure 5:**
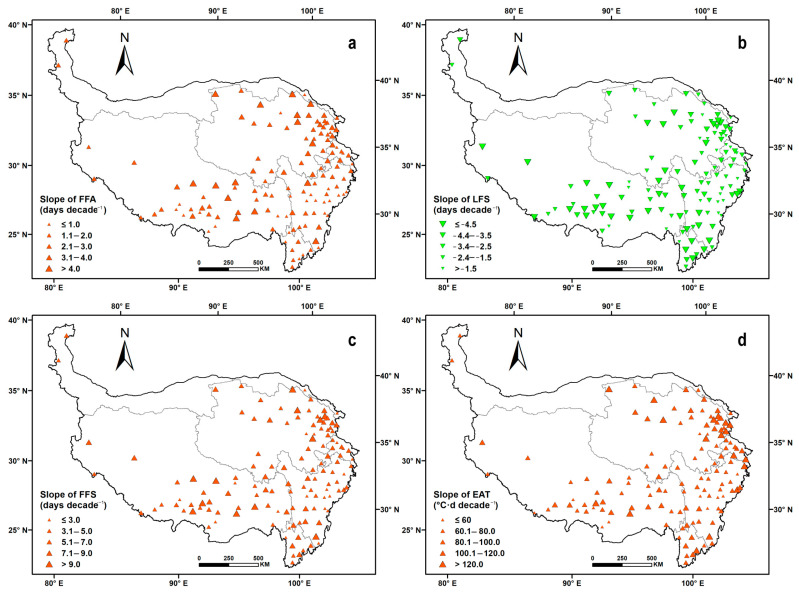
Change trends of first and last frost days and frost-free periods on the Qinghai–Tibetan Plateau during the 1978–2017 period. a–d represent the spatial distribution of annual change rates of FFA, LFS, FFS and EAT at the site level.

The FFA showed a delayed trend across all meteorological sites throughout the QTP. A larger delay rate (>4.0 days per decade) was identified at 15 sites in central Tibet and eastern Qinghai, and the largest delay rate was up to 6.2 days per decade. At 56 sites distributed in southern Tibet, northeastern Qinghai and southern Sichuan, the delay rates were between 2.1 and 4.0 days per decade. Overall, 60 sites in western and southern Tibet and central Sichuan were delayed at a rate below 2.0 days per decade; at 28 sites of Sichuan and southern Tibet in particular, it was less than one day per decade (Figure 5a). The LFS showed an advancing trend at all 131 sites throughout the QTP, and its advance rate was between 2.0 and 5.3 days, larger than the delay rate of the FFA. Spatially, the advance rate was more than 4.5 days per decade at 35 sites in Yunnan, southern Qinghai and Tibet. Lower advance rates (below 2.5 days per decade) were identified at 46 sites, which were mainly distributed in eastern Qinghai and northern Sichuan. At the other 47 sites, the advance rate of LFS was between 2.5 and 4.5 days per decade (Figure 5b).

The change rate of the FFS was between 2.8 and 11.2 days per decade on the QTP. In northern Qinghai, central Tibet and Yunnan, the change rates of the FFS at 12 sites were relatively large (more than 9.0 days per decade), induced by higher rates of FFA delay and LFS advance. The change rate was below 3.0 days per decade at 22 sites of eastern Sichuan and southern Tibet, mainly due to the lower delay rate of LFS (Figure 5c). Correspondingly, the EAT in FFS showed an upward trend throughout the QTP and its increase rate varied between 16.2 and 173.3 °C·d per decade. Spatially, the annual increase amplitude generally showed a downward trend from north to south, except for some southern sites in Tibet and Yunnan. At 35 sites in southern Tibet, northern Qinghai and Yunnan, the increase rate of EAT was mostly larger than 120.0 °C·d per decade. Sixteen sites with increase rates below 60.0 °C·d per decade were mainly concentrated in the central Sichuan and junction areas of Qinghai, Tibet and Sichuan. Furthermore, the other 80 sites were mainly between 60.1 and 120.0 °C·d per decade (Figure 5d).

### 4.3. Influence of FFS Change on Spring Wheat Potential Yield

Our previous study indicated that the regional average potential yield of spring wheat increased by 71.8 kg/ha per decade (*R*^2^ = 0.156, *p* < 0.05) on the QTP in the 1978–2017 period, and its change rate varied greatly at different altitudes, taking a 3000 m elevation as the threshold boundary [31]. In the ranges of <2000 m and 2000–3000 m, the potential yield of spring wheat decreased by 308.4 and 79.1 kg/ha per decade, while it showed an increasing trend at rates of 213.9 and 615.1 kg/ha per decade in the ranges of 3000–4000 m and >4000 m.

**Figure 6:**
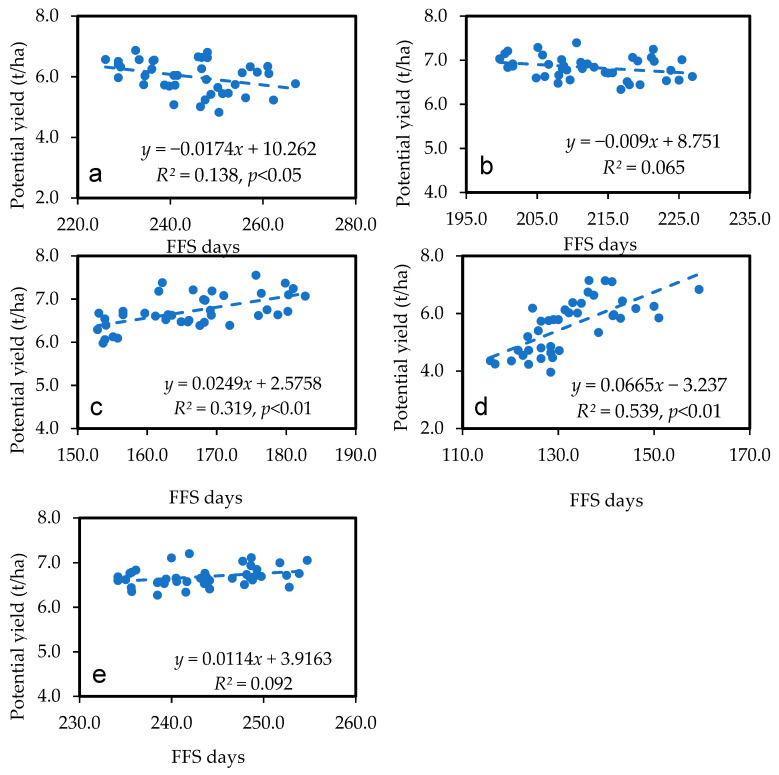
Correlation relationship between FFS and spring wheat potential yield in different altitude ranges on the QTP in the 1978–2017 period. (**a**,**b**,**c**,**d**,**e**) represent the altitude ranges of <2000, 2000–3000, 3000–4000, >4000 m and the whole QTP, respectively.

Furthermore, at different altitude ranges, the potential yield of spring wheat showed different or even opposite correlational relationships with FFS change. With increased FFS days, a significant downward trend in the potential yield was obtained in the ranges of <2000 m and 2000–3000 m, and the respective decrease amplitudes were 17.4 and 9.0 kg/ha per increased day of FFS (*R*^2^ = 0.138, *p* < 0.05; *R*^2^ = 0.065) (Figure 6a,b). However, the potential yield increased significantly with lengthening FFS days in the ranges of 3000–4000 m and >4000 m, and the respective increase amplitudes were 24.9 and 66.5 kg/ha for each prolonged day of FFS (*R*^2^ = 0.319, *p* < 0.01; *R*^2^ = 0.539, *p* < 0.01) (Figure 6c,d). For the regional average on the whole QTP, the change in the FFS had a slightly positive influence on the potential yield of spring wheat, i.e., each one-day increase in FFS would improve the potential yield by 11.4 kg/ha (*R*^2^ = 0.092) (Figure 6e).

## 5. Discussion

### 5.1. Changes in FFS and Its Driving Factors

Our results showed that the FFA and LFS on the QTP were delayed and advanced, respectively, and the advance rate of LFS was larger than the delay rate of FFA. The FFS showed a significant increasing trend (*p* < 0.05). It should be noted that the change rates of FFA, LFS and FFS are significantly dependent on the choice of research period and length. For instance, Ning et al. found that the delay rate of FFA on the QTP mostly ranged from 1.5 to 3.0 days per decade from 1951 to 2012, and the advance rate of the LFS was around 1.5 days per decade. Laba Tsering analyzed daily meteorological data obtained in the 1981–2010 period and found that the frost days in Tibet decreased at a rate between 3.3 and 14.6 days per decade [9]. Du et al. also predicted that the first frost day in the agricultural areas of Tibet continued to decrease with a rate of 1.9–9.6 days per decade from 1961 to 2010, indicating that the FFS was gradually prolonged in this area [13].

Climate warming is the main reason for the increasing length of the FFS on the QTP. Zhang and Lu indicated that the increase rate of the average temperature in the crop-growing season was between 0.01 and 0.97 °C per decade from 1978 to 2017, and the regional average was 0.32 °C per decade [31]. Furthermore, it should be noted that the increase rate of the daily minimum temperature on the QTP was 0.47 °C per decade, much larger than those of the daily maximum temperature and average temperature. Therefore, the increased daily minimum temperature could be the main cause of the delayed FFA and the advanced LFS. Considering that the advance rate of the LFS was much larger than the delay rate of the FFA, the warming trend in spring might contribute more to the lengthening of the FFS.

According to our results, the rate of the contribution of the advanced LFS on the QTP to the increase in the FFS was 60.7%, larger than that of the delayed FFA (39.3%). Furthermore, some studies also found that the length of the FFS was closely related to large-scale atmospheric circulation. For instance, Zhang et al. pointed out that the length of the FFS on the QTP was significantly negatively correlated with the Arctic Polar Vortex Index (APVI) (*p* < 0.05) [9]. Zhang et al. and Gu et al. also found that the APVI would influence climate change on the QTP and that the declining polar vortex area in the northern hemisphere was another main reason for the increased FFS [35,36].

Furthermore, our study also found that the FFA and LFS were delayed and advanced, respectively, with increasing altitude, and the FFS showed a decreasing trend, which was mainly induced by the vertical zonal rule that temperature decreases with increasing altitude. Regarding the change trend, the delay rate of the FFA, the advance rate of the LFS and the increase rate of the FFS gradually increased with increasing altitude, similar to the findings of other studies [9,10]. Tian et al. found that the change rate of frost duration at low altitudes was much higher than that at high altitudes by examining ice core records, also supporting our results [37].

### 5.2. Effects of Prolonged FFS on Agriculture

Our results showed that the changes in the number of FFS days had different effects on the potential yield of spring wheat at various altitude ranges on the QTP. In low-altitude areas, mainly below 3000 m of elevation, the EAT required by crop growth can be satisfied due to the relatively high accumulated temperatures in the crop-growing season [31,38]. Therefore, the prolonged FFS length and increased accumulated temperature shorten crop growth duration, leading to decreased spring wheat yield. However, in areas with higher altitudes, the temperature in the growing season is relatively low, which can hardly meet the EAT required for crop growth. As a result, the lower temperatures might reduce the photosynthetic efficiency of leaves, even causing frost harm to the crop body [4]. Therefore, an increased number of FFS days is conducive to meeting the EAT required for grain crop growth and the formation of matter accumulation, thus improving potential yield [39,40].

The FFA and LFS are also important indicators used to determine the start and end of the crop-growing season in alpine areas of the QTP. The delay of the FFA can reduce the possibility of frost disaster in grain crops and is thus beneficial to the safe wintering of winter wheat and highland barley, and an advanced LFS contributes to the adjustment of the sowing date of crops and improvement of their climate adaptability [8,9]. However, it should be noted that the number of FFS days calculated using the applied methodology in this study was not appropriate for frost agricultural risk assessment since a minimum temperature below 0 °C can also decrease crop yields, even if it appears for less than five consecutive days, depending on the phenological stage of the crop.

Furthermore, increased EAT during the FFS period could also increase the upper limit of altitude suitable for crop cultivation on the QTP, and thus expand the area of arable land and improve crop planting intensity [10,41]. For instance, Zhang et al. indicated that from 1970 to 2000, the upper elevation limits suitable for monoculture and double cropping on the Tibetan Plateau increased, respectively, by 4.6% and 2228.9%, and from 19,110 and 9 km^2^ to 19,980 and 2015 km^2^ [42]. Increased FFS and EAT also contribute to the lengthening of the growing period and improvement in the biomass of grassland, which could effectively increase the grazing time of herders and promote meat production from livestock [43,44].

## 6. Conclusions

It is of practical significance for optimizing the allocation of agricultural production and developing the potential crop yield to clarify the variation characteristics of FFS on the QTP. With the methodology of Sen’s Slope, GIS and correlation analysis, this study statistically analyzed the change trends of the FFA, LFS, FFS and EAT at the site level in a long time series and their influences on the potential yield of spring wheat. From 1978 to 2017, the regional average FFS and EAT on the QTP showed significant increasing trends (*p* < 0.05) induced by a delayed FFA and an advanced LFS, and the contribution of advanced LFS was larger than that of the former. The effect of climate warming on the QTP was the main reason for the lengthening of the FFS, especially the increasing trend of the daily minimum temperature, which had a larger contribution. The large-scale atmospheric circulation in the northern hemisphere might be another important reason. The prolonged FFS season had different influences on the potential yield of spring wheat when taking a 3000 m elevation as the threshold boundary. Furthermore, an increased EAT was also conducive to raising the upper limit of altitude suitable for crop cultivation on the QTP, thus expanding the arable area. Future studies should focus on exploring the influence of multiple climatic factors on crop yield based on experimental field data and model technologies to provide precise policy suggestions for regional agricultural production.

## Data Availability

The data that support the findings of this study are available from the corresponding author upon reasonable request.
